# Exposome, oxidative stress and inflammation in persons with multiple sclerosis: the EXPOSITION study protocol

**DOI:** 10.3389/fpubh.2025.1688158

**Published:** 2025-10-22

**Authors:** Maria Cristina Monti, Rachele De Giuseppe, Gloria Bertoli, Mulubirhan Assefa Alemayohu, Ghanya Al-Naqeb, Elena Ballante, Roberto Bergamaschi, Tania Camboni, Camilla Ceccarani, Chiara Ceriani, Elena Colombo, Clarissa Consolandi, Francesca Costabile, Stefano Decesari, Francesca Gallivanone, Bruno Giovanni Galuzzi, Matteo Gastaldi, Clarissa Gervasoni, Simona Gugiatti, Teresa Itri, Aliki Kalmpourtzidou, Tony Christian Landi, Aurora Lanzotti, Alessia Lo Dico, Federica Loperfido, Beatrice Maccarini, Antonio Mazza, Cristina Montomoli, Enrico Oddone, Noemi Paulin, Chiara Pellizzer, Roberta Pernetti, Anna Scarabotto, Francesca Sellaro, Marco Severgnini, Donato Summa, Gemine Vivone, Danilo Porro, Hellas Cena, Eleonora Tavazzi

**Affiliations:** ^1^Unit of Biostatistics and Clinical Epidemiology, Department of Public Health, Experimental and Forensic Medicine, University of Pavia, Pavia, Italy; ^2^Laboratory of Dietetics and Clinical Nutrition, Department of Public Health, Experimental and Forensic Medicine, University of Pavia, Pavia, Italy; ^3^Istituto di Bioimmagini e Sistemi Biologici Complessi (IBSBC), National Research Council (CNR), Milan, Italy; ^4^IRCCS Mondino Foundation, Pavia, Italy; ^5^Institute for Biomedical Technologies, National Research Council (ITB-CNR), Segrate, Italy; ^6^Institute of Atmospheric Sciences and Climate-Italian National Research Council (ISAC-CNR), Rome, Italy; ^7^Institute for Atmospheric Sciences and Climate, National Research Council of Italy, Bologna, Italy; ^8^Institute of Methodologies for Environmental Analysis, CNR-IMAA, Tito Scalo, Italy; ^9^Unit of Occupational Medicine, Department of Public Health, Experimental and Forensic Medicine, University of Pavia, Pavia, Italy; ^10^Hospital Operative Occupational Medicine Unit, ICS Maugeri IRCCS, Pavia, Italy; ^11^Unit of Clinical Nutrition, ICS Maugeri IRCCS, Pavia, Italy

**Keywords:** multiple sclerosis, exposome, air quality, lifestyle, nutritional status, microRNA, neurofilaments, inflammation

## Abstract

The exposome represents the totality of external and internal exposures an individual encounters throughout life and plays a critical role in developing many chronic diseases, including multiple sclerosis (MS). MS is a multifactorial disease influenced by both genetic and environmental factors. The EXPOSITION study (registered on www.clinicaltrials.gov, NCT06325358) aims to investigate the association between environmental exposures (external exposome) and biological markers of oxidative stress and inflammation (internal exposome) in people with MS. This cross-sectional study will involve 200 individuals with MS, assessed for lifestyle and occupational variables and biological markers, including circulating microRNAs, neurofilament light chains, pro- and anti-inflammatory cytokines, and gut/nasal microbiota composition. The study will use advanced statistical models, such as generalised linear models and multivariate analyses, to assess associations between external exposures and biological outcomes. By integrating both environmental and biological factors, this research aims to deepen our understanding of MS mechanisms, providing insights that could lead to targeted interventions, personalised therapies, and public health strategies to mitigate MS progression.

## 1 Introduction

Neurological disorders pose a global health challenge (1). They have shown the greatest percentage increase in disability-adjusted life years (DALYs) across all ages and constitute a significant proportion, accounting for the largest percentage change in late-life deaths among non-communicable diseases (NCDs) in the last 10 years (1). Among neurological diseases driven by a degenerative mechanism, Multiple sclerosis (MS) is the most common autoimmune and neurological disease of the central nervous system (CNS), characterised by demyelination and neuroinflammation (2, 3). MS is growing alarmingly worldwide, as 2.8 million people were reported living with MS in 2020, corresponding to a global prevalence of 35.9 per 100,000 population (30% increase over 2013) (4), probably in part related to more sensitive diagnostic criteria and easier access to healthcare facilities. Specifically, Italy in 2019 experienced a substantial increase in the age-standardised incidence of MS (2.8 per 100,000 population at risk), with a percentage change of 26.3% compared to 1990 (5). Consequently, the result is an increase in the consumption of healthcare resources of 4.8 billion euros due to MS in Italy (6, 7). In the past 5 years, the identification of new genetic factors and significant evidence for the role of environmental risk factors and gene–environment interactions has led to a greater consensus about its multifactorial nature (8). Whether MS inflammation is in response to a chronic viral infection (9), primary neurodegenerative processes, or a reflection of a dysfunctional immune system is still debated (8). Nevertheless, inflammation is a major driver of pathology and oxidative stress (OS), which is heavily involved in several MS pathological hallmarks such as myelin destruction and axonal degeneration, contributes to tissue injury and promotes existing inflammatory response (10). However, such a disease, in which multiple factors are involved in its pathogenesis, requires a comprehensive understanding of the role of these factors in explaining the variation in biomarkers of OS and inflammation that underline the pathophysiology (8, 10).

Several environmental factors can influence pro-oxidant and pro-inflammatory pathways that impact human health throughout life. According to the periods of heightened susceptibility and multiple risks, lifestyle factors (e.g., dietary habits, stress, physical activity, smoking, toxic chemicals exposure, and social condition) and environmental ones (e.g., air pollution, green space, noise, light, and traffic) both contribute to the exposome, which can drive human health towards health or disease (11).

Exposome was defined for the first time in 2005 as a new concept to identify and study the totality of environmental exposures individuals experience over their lifetime and how these exposures relate to health outcomes (12). It describes the harmful biochemical and metabolic changes that occur in our body due to the totality of different environmental exposures throughout the course of life, ultimately leading to adverse health effects and premature deaths (12). Exposome includes three domains: (i). a general external environment (e.g., air pollution, urban environment and climate); (ii). a specific external environment (e.g., physical activity, nutrition and other lifestyle habits, as well as social-economic determinants); (iii). an internal environment, which refers to exposures inside the body that are unique to the individual (e.g., metabolic processes, circulating biomarkers, hormones) (13).

Several studies have examined the relationship between exposome variables and MS (14, 15).

Among the most extensively studied environmental factors associated with MS, external exposures such as Epstein–Barr virus infection, insufficient levels of 25-hydroxyvitamin D [25(OH)D] due to limited sun exposure, tobacco smoking, and early-life obesity have been identified as significant contributors (14).

Pollution has also been recognised as a potentially critical factor affecting the CNS through diverse mechanisms and should be included as an external exposome variable (15). Exposure to air pollution, particularly traffic-related air pollution, can induce several potential mechanisms, including inflammation and OS, particle translocation directly to target organs such as the brain, spillover into circulation, oral intake leading to a change in gut microbiome, and neurohormonal activation (16). Among these, OS mechanisms have been largely considered in literature (11, 17, 18).

Recently, the Health Effects Institute (HEI) has suggested that arguably only OS is seemingly a realistic possibility in the low air pollutant concentration context, which is now characterizing many high-income countries (18).

Traffic-related air pollution is a well-established environmental risk factor, particularly in urban areas (11). Chronic or long-term exposure to ambient air pollution, particularly traffic-related air pollution, and to the fine fraction of ambient particulate matter (PM2.5, with aerodynamic diameter <2.5 μm), gaseous pollutants, and heavy metals have been shown to elevate levels of neuroinflammatory and pro-inflammatory markers in the human brain (19). PM2.5, particularly its ultrafine fraction (also called UFP or PM0.1, indicating particles with a diameter less than 0.1 μm), has been reported as a risk factor for neurodegenerative diseases (20–22). These tiny particles (PM2.5 and PM0.1) pose a significant health hazard due to their ability to penetrate the bloodstream, inducing inflammation and OS within the systemic vasculature (11). UFPs’ smaller particle size enhances their penetration capacity, prompting growing interest in these tiny particles as a potential contributor to adverse health effects (21). For example, we recently reported that even single exposures to urban UFPs can be a significant source of OS and inflammation consistent with a Trojan-horse mechanism for nanoparticles associated with toxic constituents (23). These inflammatory and oxidative processes may result in neuroinflammation, neurodegeneration, and blood–brain barrier disruption, which appear to be associated with the onset and relapses of MS (24).

Additional external exposome factors, such as climate anomalies and extremes, poorly designed urban environments (e.g., limited green spaces), unhealthy lifestyle behaviours (such as pro-inflammatory diets or inadequate physical activity), occupational exposures, and psychosocial stress, have also been implicated in the broader burden of inflammatory diseases (11).

Evaluation of the exposome is challenging due to the necessity of collecting exposure data across different variables (13). The most comprehensive and novel approach is the functional exposome, which examines the relationship between the sum of environmental exposures (external exposome) and the biological effects they cause (internal exposome). Therefore, nowadays, exposome science operates at the -omics scale, employing high-throughput technologies to analyse biological samples and environmental factors, leading to the simultaneous examination of various molecular components, including genomics, transcriptomics, proteomics, and metabolomics (25). By adopting these omics approaches, exposome research extends beyond traditional assessments of isolated exposures, enabling the holistic exploration of the interplay between environmental influences and biological responses, also in MS (25).

Thus, it is essential to improve the understanding of the extent to which external exposome is associated with MS, which would potentially result in the introduction of precision medicine based on new biomarkers for more efficient disease management and slower disease progression.

Consequently, a study protocol was designed to conduct a cross-sectional study on people with Multiple Sclerosis (pwMS), aimed to understand how biological biomarkers of OS and inflammation vary in response to the external exposome in pwMS. To the best of our knowledge, this is the first study that analyzes how the external exposome influences the internal exposome in pwMS, taking into consideration multiple exposome variables belonging to the general and specific external exposome, as well as the internal one.

## 2 Materials and methods

### 2.1 Objectives

**Figure 1:**
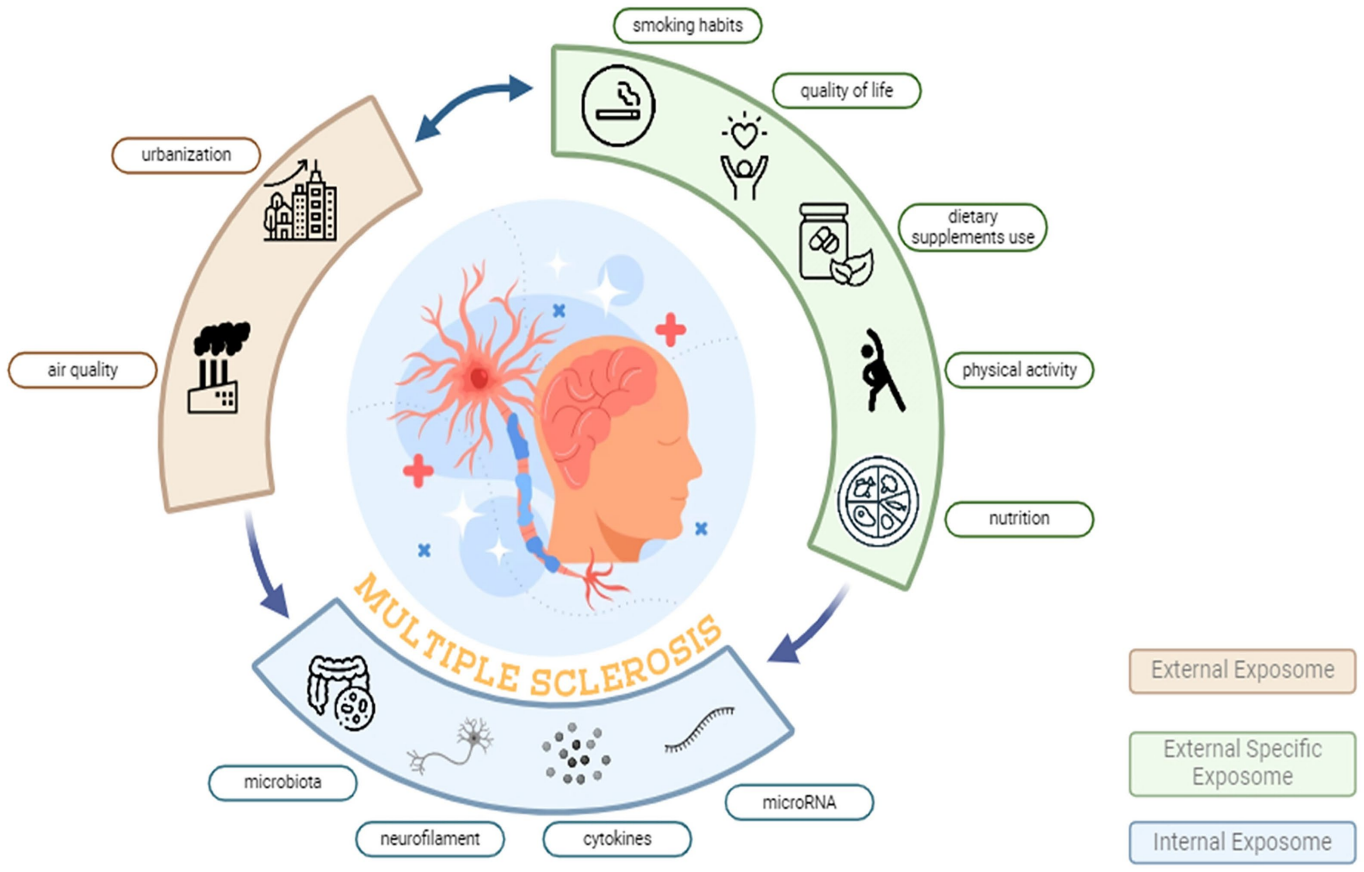
External exposome and biological responses in multiple sclerosis: the EXPOSITION approach. The diagram depicts the urban exposome domains hypothesized to influence MS. The External Exposome (beige arc) summarises the broad environmental context including urbanization and air quality typical of Po Valley settings. The External Specific Exposome (green arc) represents individual-level lifestyle and behavioural factors, including smoking habits, quality of life, dietary supplement use, physical activity, and nutrition. The Internal Exposome (blue arc) comprises measurable biological mediators and effect markers, including gut microbiota, neurofilament, circulating cytokines, and microRNA. Bidirectional arrows indicate dynamic interactions across domains, converging on MS pathophysiology (central illustration).

EXPosome, Oxidative Stress and InflammaTION in Persons with MS (EXPOSITION) study is a cross-sectional research initiative, designed to evaluate the variation of candidate biological biomarkers of OS and inflammation in response to the external exposome in pwMS (Figure 1).

Particularly, the primary objective of the EXPOSITION study is to investigate the variation of microRNA (miRNA) expression, in response to general external exposome (urban air pollution and urbanization), as well as occupational and lifestyle external exposome components in pwMS, controlling for other validated biomarkers (e.g., serum pro- and anti-inflammatory cytokine levels, serum neurofilaments levels, serum vitamin D levels), gender, age, anthropometric measurements, and medical history.

The secondary objective is to investigate the variation of the gut and nasal microbiota composition as candidate biological biomarkers of OS and inflammation, in response to the exposome components and other variables included in the primary objective methodological framework. The microbial profiles will be evaluated in terms of (i). Biodiversity (alpha-diversity), estimating the number of taxa in each sample and statistically comparing estimates across the experimental groups; (ii). Composition (beta-diversity), evaluating the pairwise similarity among the samples and clustering them according to their distances; (iii). Bacterial groups (relative abundances), evaluating whether a bacterial taxon is significantly increased or depleted in pwMS belonging to the different exposome profiles under investigation.

### 2.2 Population, inclusion criteria and informed consent

To address EXPOSITION’s purposes 200 persons with MS attending the Multiple Sclerosis Center of the IRCCS Mondino Foundation, Pavia (Italy) have been selected and recruitment is under way for this study, according to the following inclusion criteria: age ≥18 years; diagnosis of MS according to 2017 McDonald criteria (26); relapsing–remitting (RR) MS, which is the most common MS type and is characterized by alterations of relapses and remission periods (7); routine clinical examinations scheduled in the study period March–April, 2024 and October–April, 2025; Residence in the provinces of Pavia or Milan (Italy); informed consent form signed. Particularly, the study will be conducted in Pavia and Milan, the Northern Italy nodes of the National Biodiversity Future Center (NBFC) (27), which is an Italian research consortium integrating universities, institutes, and stakeholders to study, monitor, and restore biodiversity and to translate evidence into policies, technologies, and nature-based solutions for human and ecosystem health. Thus, Milan represents a large metropolitan and industrial hub with high traffic density, complex socio-economic gradients, and sustained environmental pressures, while Pavia is a medium-sized university city with peri-urban and agricultural interfaces and substantial daily commuting flows to Milan. This paired selection will capture heterogeneous but complementary northern urban exposome profiles within a shared atmospheric basin typical of the Po Valley and characterized by frequent stagnation events.

People refusing to participate in the study will be excluded. Potentially eligible pwMS will be screened by a neurologist, an expert in MS, who will verify that all the inclusion criteria will be fulfilled.

Participants will be selected from the Pavia Database of Multiple Sclerosis of the Multiple Sclerosis Center of the IRCCS Mondino Foundation, Pavia (Italy). It includes 1,400 MS patients, has been active since 1990 and has been part of the ‘Italian Multiple Sclerosis Register’ since 2015.

The study will follow the Declaration of Helsinki. Written informed consent will be obtained from the participants.

Ethical approval was granted by the Ethical Committee of IRCCS Policlinico San Matteo (Pavia) (protocol number: 0040023/23; Accepted: 25/07/2023). The EXPOSITION protocol has been registered on www.clinicaltrials.gov (NCT06325358).

### 2.3 Variables collection

**Figure 2:**
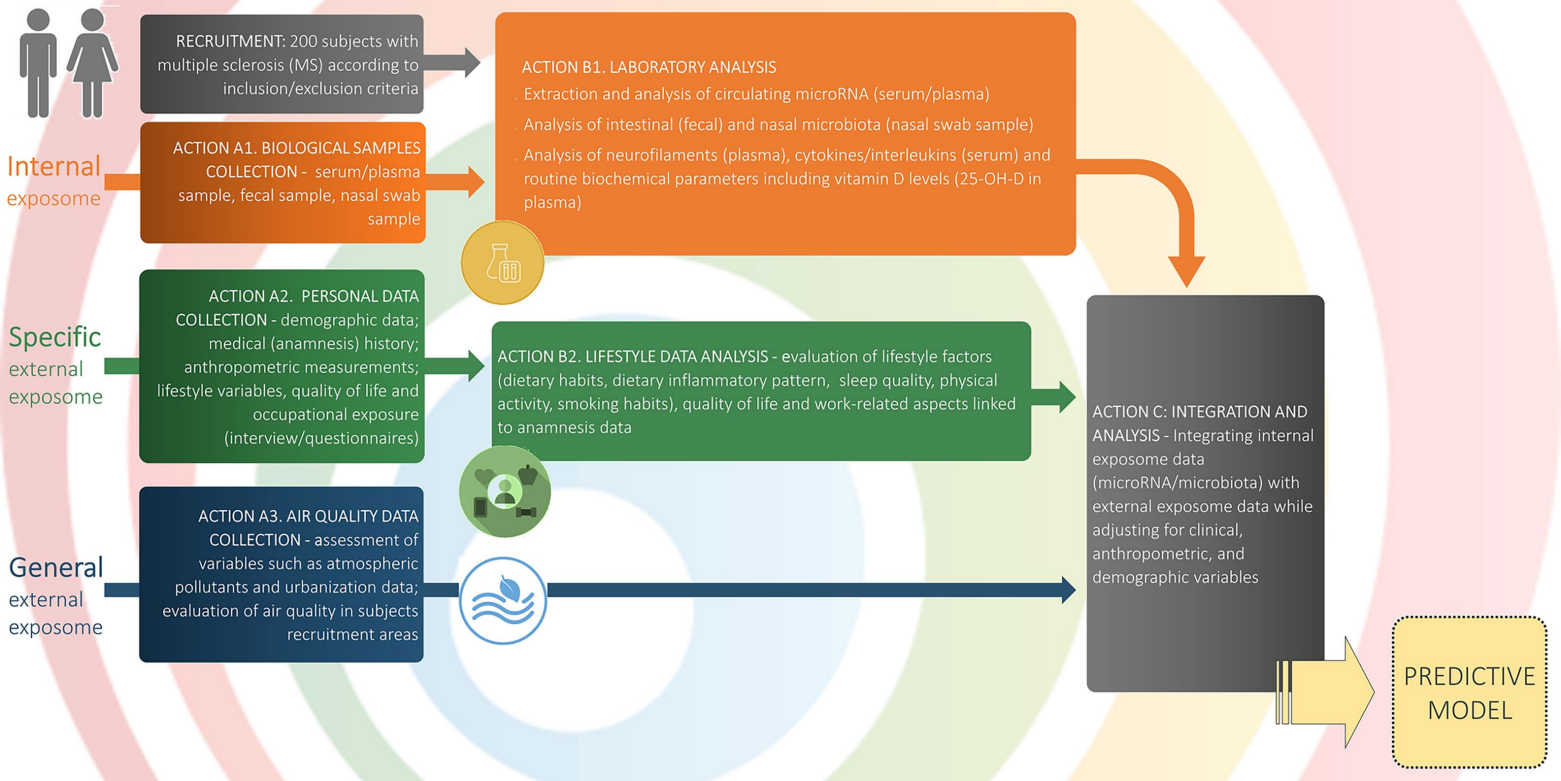
Study workflow and data collection. The schematic outlines participant flow, data domains, and analytic steps. Recruitment: 200 adults with MS meeting inclusion/exclusion criteria. Internal exposome (orange): Action A1: biospecimen collection (serum/plasma, feces, nasal swab); Action B1: laboratory assays of circulating microRNA (serum/plasma), intestinal (stools) and nasal microbiota (nasal swab), neurofilaments (plasma), cytokines/interleukins (serum), and routine biochemistry including 25-OH-vitamin D. Specific external exposome (green): Action A2: personal data collection (demographics, medical history, anthropometry, lifestyle variables, quality of life, occupational exposure via interviews/questionnaires); Action B2: lifestyle data analysis (dietary habits and inflammatory pattern, sleep quality, physical activity, smoking, quality of life, and work-related factors). General external exposome (blue): Action A3: air-quality and urbanization metrics in recruitment areas (e.g., atmospheric pollutants; area-level indicators). Integration and analysis (grey): Action C: multivariate integration of internal and external exposome data with adjustment for clinical, anthropometric, and demographic covariates, yielding a predictive model (yellow). This schematic mirrors the Statistical Analysis section. Arrows denote the directional flow from data collection to analysis and model construction.

All participants will be investigated for social, demographic and clinical data as well as general and specific external exposome factors (Figure 2). The internal exposome factors, including the primary and secondary endpoints of the study, will be collected for each participant from blood and stool samples, as well as from nasal swabs. PwMS will also be assigned to a geographic unit, according to their municipality of residence. Therefore, air pollution and urbanisation will be collected as ecological characteristics related to their geographic unit of origin.

#### 2.3.1 Social, demographic and clinical data

Social and demographic data includes age, sex, ethnicity, date and place of birth, civil status, education level, employment status, and current residence address. Clinical data includes information on disease onset and diagnosis (date of onset, date of diagnosis, presence of oligoclonal bands), disease duration, MS phenotype, date of last relapse, information on the most recent MRI (new/enlarging T2-weighted lesion, Gadolinium enhancing lesions) and No-evidence of disease-activity status (NEDA3), information on disease-modifying treatments (DMT-number and type of previous treatments, and reasons for discontinuation, start date of the ongoing treatment) as well as of symptomatic treatments related to MS or any other clinical condition. The level of clinical disability will be quantified using the Expanded Disability Status Scale (EDSS) (27). All the data will be anonymised and collected in RedCAP.

#### 2.3.2 General and specific external exposome factors

##### 2.3.2.1 Air quality evaluation

Two-dimensional and three-dimensional fields of major atmospheric components calculated by state-of-the-art modelling systems and acquired from satellite remote sensing observing systems will be provided.

Both CAMS (ensemble forecasting system developed and operated by Copernicus Atmosphere Monitoring Service) and CHdioxide, a highly transport model developed by CNR-ISAC on the BOLAM meteorological model air quality models will provide quantitative spatial information—with a horizontal resolution spanning between 8 and 10 km—on ambient PM and gases mass concentrations, such as PM10 (PM having an aerodynamic diameter smaller than 10 μm), PM2.5 (PM having an aerodynamic diameter smaller than 2.5 μm), BC (black carbon, a fraction of carbonaceous aerosols highly responsible for the absorption of the visible light and forming from combustion processes), NO_2_ (nitrogen dioxide, highly reactive gas, emitted mainly by traffic and other sources in the transport sector), and O_3_ (ozone, highly reactive gas). Hourly, daily, weekly, and seasonal concentrations of these air pollutants for the period of interest (since March 2022) will be considered to characterise the air quality for Milan and Pavia provinces.

Furthermore, for the same period and over the same areas, Sentinel-5P gaseous air pollutants products, generated from the joint use of both radiances acquired by the TROPOspheric Monitoring Instrument (TROPOMI) sensor (a hyperspectral sensor capturing around 4,000 spectral bands with high temporal resolution and 3.5 km x 5.5 km spatial resolution at nadir) and ancillary data, will be included as gridded emissions with 3 km x 3 km spatial resolution (possibly resampled to a 1 km x 1 km grid) for the whole time series (daily values). More specifically, tropospheric total columns of O_3_, SO_2_ (sulfur dioxide), NO_2_, and CO (carbon monoxide) Level 2 products will be distributed. Pollutant-dependent post-processing based on mathematical morphology or nonlinear filtering is performed to fill missing data gaps or reduce noise by preserving relevant information.

These data will be associated with municipalities belonging to the two provinces, exploiting a nearest-neighbour approach based on the geographic position of each pwMS derived from the residence address (geo-positioning). Air pollution data will be analysed to apportion major emission sources and their processing in the atmosphere. A source apportionment analysis using chemical transport modelling will be performed on the data to assess the contribution of urban (traffic-related) air pollution to overall exposure. Additional information about personal exposure to specific sources from questionnaires will be considered, including specific questions aimed at quantifying the daily time spent indoors/outdoors and close/far from the residence address.

##### 2.3.2.2 Urbanization

The European degree of urbanisation classification (28) based on 2020 census results will be adopted to stratify territories according to urbanisation. Municipalities in the provinces of Pavia and Milan will be classified as cities, town-suburbs or rural areas.

The municipalities classifiable as cities by DEGURBA (28) will be only Pavia in its province, Milano and 47 other territories in the Milan province. Moreover, the territories classifiable as town-suburbs will be 27 in Pavia and 77 in Milano. Pavia province will have 158, and Milano 8 rural areas. DEGURBA uses country-specific definitions of what a city is. In Italy, according to the ISTAT definition, a city (or urban area) is defined as a local administrative unit with a minimum of 50,000 inhabitants, where most of the population lives in a high-density cluster.

##### 2.3.2.3 Nutritional status and lifestyle assessment

The assessment of the nutritional status of study participants will include both instrumental measurements to evaluate body weight and composition, as well as analyses of dietary intake. These will involve examining eating habits and food consumption frequencies using specifically validated questionnaires.

Particularly trained personnel will conduct anthropometric and body composition measurements under standardised conditions. Body weight (kg) will be measured with participants in their underwear, using a calibrated balance scale with an accuracy of ±100 g. Height (cm) will also be recorded, and the Body Mass Index (BMI) will be calculated as weight (kg) divided by height (m) squared (29).

Waist circumference (WC) will be measured to the nearest centimetre using a flexible steel tape with participants standing upright, arms crossed on opposite shoulders, and after a gentle exhalation. The measurement will be taken at the midpoint between the lowest rib and the upper lateral margin of the right iliac crest. The waist-to-height ratio (WHtR), an established marker of abdominal adiposity with a cut-off of <0.5 (30), will also be determined.

Body composition, including phase angle, will be assessed using bioelectrical impedance analysis (BIA) with the BIA 101 BIVA® PRO device (31). Phase angle, a reliable indicator of cellular health and nutritional status (32), will be analysed alongside other parameters to provide a comprehensive evaluation of participants’ body composition (33).

Regarding lifestyle habits, the trained personnel will also collect lifestyle variables, using previously validated questionnaires (34–38): dietary habits and frequency of consumption; physical activity level; smoking habit; quality of sleep; level of stress and QoL. The dietary habits of the participants will be evaluated with the use of a validated food frequency questionnaire (FFQ) adapted to the Italian population (36) and adjusted based on current food trends, such as the consumption of plant-based milk and meat. Detailed information about the daily food consumption of pwMS will be gained through a 24-h dietary recall.

Dietary supplement use will be assessed based on the type and nutrients included in the dietary supplements, their dose and their frequency of use.

The Dietary Inflammatory Index (DII) will also be used to quantify the overall effect of diet on inflammatory potential. The DII will be calculated based on the daily food consumption data of the participants to calculate the food parameters included in the index, such as vitamins, minerals, green/black tea consumption and others (39). The scoring algorithm ranges from −1, which represents a maximally anti-inflammatory factor, to +1, which represents a maximally pro-inflammatory factor. Finally, all food parameters are summed to the overall DII score for the diet of everyone (39).

The MEDI-LITE score will be used for the assessment of adherence to the Mediterranean dietary model (37). This scoring system is based on a questionnaire, which investigates the frequency of consumption of nine classes of food; the final score varies from 0 (low adherence) to 18 (high adherence); overall, subjects who obtain a score higher than 9 will have a significantly increased adherence to the Mediterranean Diet (37).

Physical activity levels will be evaluated by using the short form of the International Physical Activity Questionnaire (IPAQ) (35), which includes 7 items concerning the last 7 days’ levels of moderate, vigorous and sedentary physical activity The metabolic equivalent of tasks (MET) will be calculated based on the frequency and duration of physical activity and expressed as MET-minutes per week (MET-min/week) (35). The calculation will follow the formula: METs = MET level × minutes of activity × events per week (35).

Based on the total MET score, participants will be classified into three categories of physical activity: low, moderate, and high (35). Individuals will be considered to have a low level of physical activity if they do not meet the criteria for the moderate or high categories (35). The moderate activity level will include participants who reported vigorous-intensity activity on at least 3 days for a minimum of 20 min per day; or moderate-intensity activity and/or walking on at least 5 days for a minimum of 30 min per day; or any combination of walking, moderate- or vigorous-intensity activities on five or more days, reaching a total of at least 600 MET-minutes per week (35). The high activity level will comprise individuals who reported vigorous intensity activity on at least 3 days and accumulated a minimum of 1,500 MET-minutes per week, or any combination of walking, moderate-, or vigorous-intensity activities performed on seven or more days, achieving a total of at least 3,000 MET-minutes per week (35).

Sleep quality will be measured by the Pittsburgh Sleep Quality Index (PSQI) questionnaire (34), consisting of 9 multiple-choice items divided into 5 different components related to subjective sleep quality, sleep latency, sleep duration, habitual sleep efficiency, sleep disturbances, use of sleep medication and daytime dysfunction. The global PSQI score will be obtained by considering the scores of all the components, and it ranges from 0 to 13. The PSQI cut-off of 5 or above indicates poor sleep quality (34).

Cigarette smoking habits will be documented by classifying participants as current smokers, former smokers, or non-smokers. For current smokers, the number of cigarettes smoked per day and the duration of smoking in years will be recorded. Former smokers will be asked to report the number of years they smoked and their average daily cigarette consumption during that period.

##### 2.3.2.4 Quality of life

QoL will be evaluated by Multiple Sclerosis Quality of Life-29 (MSQOL-29) (38), a multidimensional health-related QoL measure that combines both generic and MS-specific items into a single instrument. The questionnaire consists of 29 items, divided into 7 subscales related to physical functioning, pain, emotional well-being, energy, cognitive function, health stress, sexual function, change in health, social function, health perception and overall QoL (38).

##### 2.3.2.5 Occupational factors

The potential exposure to harmful agents in the workplace will be assessed through a specific questionnaire aimed at identifying the exposure to the entire working life of the subjects involved. The information collected regarding the duration of each work activity and the tasks performed throughout work life will be investigated. This will include the specific sector(s) of employment, the main tasks performed, and the workplace location(s). Following this information, the related International Standard Classification of Occupation (ISCO) code will be assigned (40). The ISCO code will be adopted to determine the level of occupational risk for SM of the involved subjects, according to four classes: (1) work activities associated with specific occupational risks connected with SM (such as agricultural workers, offshore workers, and hairdressers (41); (2) work activities associated with occupational risks less likely connected with the SM diagnosis (such as exposure to toxic oil well fumes, low-frequency magnetic fields, and pesticides (41); (3) work activities not directly connected with increased risk for SM, but identified as potential causes of relevant professional diseases through inflammatory pathways (e.g., exposure to carcinogenic and mutagenic agents, toxicants, ionizing radiation, etc.); (4) work activities not associated with specific relevant occupational diseases (e.g., office workers, trade workers, unemployed). In the case of multiple activities during working life, the prevalent exposure, namely the one correlated with the longest working period of the subject, will be considered.

#### 2.3.3 Internal exposome variables (including the endpoints of the study)

Blood samples will be analysed to evaluate miRNA expression, neurofilament proteins, levels of pro- and anti-inflammatory cytokines, and vitamin D levels. Samples will be collected by trained research nurses without requiring participants to fast. Each sample will be anonymised using a progressive code, accompanied by the participant’s sex and date of birth. The anonymised samples will then be treated for pre-analytical processing, including centrifugation, separation, and storage.

During the pre-analytical phase, the absence of hemolysis is required for sample suitability; any hemolysis detected must be documented. Additionally, samples must be maintained at room temperature until the separation process is complete.

For the analysis of miRNA expression and vitamin D levels, and other biochemical parameters, blood specimens will be collected in tubes designed for specific serum samples (four 3.5 mL tubes without additives) and plasma samples (three 3.5 mL tubes containing either EDTA or citrate to prevent coagulation).

Plasma samples will be obtained by centrifugation of the EDTA- or citrate-containing tubes. Both serum and plasma samples will be centrifuged at 3,000 rpm for 15 min and processed within 2 h of collection. The processed samples will then be divided into aliquots (eight for serum and six for plasma), each with a minimum volume of 500 μL, and stored at −80°C until analysis.

The miRNA expression, as well as the neurofilament protein (NfL), cytokine, and levels of 25(OH)D vitamin measurements, will be assessed.

Stool samples will be collected to analyze the intestinal microbiota and will be collected by the PwMS after they are accurately instructed by a member of the research team on how to collect, store, and transport samples, according to the following inclusion criteria: (i) absence of fever and gastroenteritis; (ii) absence of diarrhea for more than 24 h in the previous 7 days; (iii) no antibiotic treatment in the previous 7 days. Similarly, the nasal swab samples will be collected using flexible flocked swabs and conserved in specific tubes containing transport medium, making them compatible with downstream sequencing assays.

Stool and nasal samples will then be anonymised by the trained dietitians and biologists who received them, using a progressive code and stored at −80 °C, until the analysis.

##### 2.3.3.1 MicroRNAs expression levels (primary endpoints)

The differential expression of a set of 5 specific circulating plasma miRNAs from the blood of each pwMS will be measured. The set of miRNAs has already been selected based on their significantly deregulated expression in MS, as evidenced in a bioinformatic analysis conducted using several public microarray databases from Gene Expression Omnibus (GSE61741, GSE31568, GSE17846, GSE21079, GSE74579, GSE215450).

We focused on those miRNAs showing the most coherence in terms of regulation direction (i.e., statistically significant up or down-regulation) across the databases. The analysis allowed us to obtain a list of miRNAs that were consistently up- or down-regulated in the comparison between MS and healthy samples. In particular, plasma samples of our cohort were analysed to quantify the level of expression of miR-30a-5p, miR-146a-5p, miR-664, miR-330, and miR-574-3p normalised on U6 expression. Notably, three miRNAs (miR-30a-5p, miR-146a-5p, miR-664) were up-regulated in four databases, and two miRNAs (miR-330, miR-574-3p) were down-regulated in three databases. Interestingly, miR-146a (42), miR-30a (43), miR-330 (44), and miR-574 [0.1002/ana.23880] have already been associated with MS.

Moreover, they have a key role in characterising inflammation and oxidative stress. In particular, miR146a acts as a negative regulator of the NFκB pathway, modulating pro-inflammatory cytokines, and has been shown to influence reactive oxygen species (ROS) production in various pathological conditions (45). miR30a also plays a crucial role in regulating inflammatory factors and oxidative stress factor expression by targeting Neurod 1 and the MAPK/ERK pathway (46). The binding of miR-574-5p to TLR8/7 triggers the TLR signalling pathway, leading to the induction of interferons, inflammatory cytokines and autoimmune signalling. These findings suggest that miR-574-5p is not only an important epigenetic regulator of gene expression, but also an important regulator of immune and inflammatory responses (47). Finally, miR330 is associated with the inflammatory responses and oxidative stress mediated by miR-330-5p/EPHB3 axis in neuroinflammation and cognitive dysfunction (48).

A portion of serum samples will be collected and immediately frozen at −20 °C. After being transported, serum samples will be thawed, and the circulating RNAs will be extracted (Trizol, Thermo Fisher Scientific). After a step of miRNA-specific reverse transcription (TaqMan miRNA reverse transcription kit, Thermo Fisher Scientific; Mir-X miRNA First-Strand Synthesis Kit, Takara), the copy-DNA obtained will be used as the template for Real Time-qPCR analyses of selected miRNAs by Singleplex TaqMan miRNA assay reactions and/or in SybrGreen-based RTqPCR (SIC).

The method of Livak et al. (49) will be applied for the comparative analysis of the relative expression of the selected miRNAs in serum samples, considering different combinations of the external exposome variables.

Specifically, RT-PCR returns the number of cycles that the samples underwent before they were detected, reported as a value known as the Cycle Threshold (CT). The CT values vary logarithmically with expression levels. ΔCT values are calculated by subtracting the CT value of the endogenous control for a given sample (or the mean of the CT values of the endogenous controls if more than one exists) from the CT value of the gene for the given sample. The ΔΔCT is calculated by subtracting the ΔCT of an experimental sample from a control sample. Fold change is calculated by raising 2 to the power of the negative ΔΔCT value since CT values are related to the amount of miRNA or gene logarithmically. A statistical analysis will be applied to highlight the differences in the expression of each miRNA in the two populations.

##### 2.3.3.2 Gut and nasal microbiota analysis (secondary endpoints)

The stool and nasal samples will be stored at −80 °C until genomic DNA extraction. The Maxwell® RSC Faecal Microbiome DNA Kit and Maxwell® RSC Pathogen Total Nucleic Acid Kit will be employed for DNA isolation from stool and nasal samples, respectively, using a Maxwell RSC automated extractor (Promega s.r.l., Milan, Italy), according to the manufacturer’s recommendations. The quality and quantity of gDNA will be assessed by TapeStation 4,200 system (Agilent, Santa Clara, CA, USA) and Qubit 4 fluorometer (Invitrogen, Waltham, MA, USA). Afterwards, the sequencing libraries will be prepared by using the MICROLAB STARlet NGS UV automated liquid handling station (Hamilton Italia Srl, Agrate Brianza, Italy), following an NGS metabarcoding approach. The 16S Metagenomic Sequencing Library Preparation protocol will be employed for the amplification of the V3-V4 hypervariable regions of the 16S rRNA gene and indexing by dual Nextera® XT indexes (Illumina, San Diego, CA, USA). All libraries, quantified by the Qubit 4 fluorometer and pooled at equimolar concentrations, will subsequently be sequenced using the Illumina NextSeq1000 platform through a 2x300bp paired-end run.

Raw sequencing reads will be processed following a specific pipeline, involving the merging of two paired reads overlapping, generating a single fragment covering the whole V3-V4 regions, the trimming and filtering of low-quality reads and the grouping to Amplicon Sequence Variants (ASVs) by the DADA2 denoising pipeline (50). Taxonomic classification will be performed by the RDP classifier (51) against the latest release of the SILVA reference database (52).

Biodiversity will be estimated by calculating the alpha-diversity for each sample using different metrics, such as Shannon’s entropy index, observed species, Chao1 index and Faith’s phylogenetic diversity index. Pairwise similarity between microbial profiles will be assessed using the unweighted and weighted implementations of the UniFrac distance (53). Microbial composition will be expressed as the relative abundance of taxa at different levels, from phylum to genus. Due to the lower complexity of the nasal microbiota, its composition will be characterised down to the species level for some of the most abundant genera (e.g., *Corynebacterium, Moraxella, Haemophilus*).

##### 2.3.3.3 Neurofilament proteins and pro-and anti-inflammatory cytokine levels assessment

Human neurofilament light chain protein (NfL) levels will be quantified from plasma samples using the Simple Plex assay (ProteinSimple, California, USA) on the Ella microfluidic immunoassay platform (ProteinSimple, California, USA), following the manufacturer’s protocol (54). The Ella device will be calibrated using the in-cartridge factory standard curve, and plasma samples will be prepared with a 1:2 dilution in the provided Sample Diluent (ProteinSimple, California, USA) (54).

Each sample will be analysed in a single well, as the Simple Plex platform automatically performs triplicate assays within the microfluidic system (54). The lower limit of quantification (LLOQ) for this assay is 2.70 pg./mL, and the upper limit of quantification (ULOQ) is 10,290.00 pg./mL (54).

Serum concentrations of various cytokines and growth factors, including interleukin (IL)-1β, IL-2, IL-4, IL-6, IL-10, IL-12, IL-17, IL-22, and IL-23, as well as transforming growth factor beta (TGF-β), granulocyte-macrophage colony-stimulating factor (GM-CSF), tumour necrosis factor-alpha (TNF-α), and interferon-gamma (IFN-γ), will be measured. These cytokines will be quantified in picograms per millilitre (pg/mL) using the Simple Plex assay (R&D Systems) and commercial enzyme-linked immunosorbent assay (ELISA) kits, including CXCL10 measurements where applicable (55).

##### 2.3.3.4 Vitamin D levels assessment

For each patient, serum 25(OH)D concentrations will be measured using the Elecsys® Vitamin D total III assay (Roche Diagnostics, Mannheim, Germany) on a Cobas Pure integrated analyser (Roche Diagnostics). The assay is an electrochemiluminescence competitive binding immunoassay designed for the quantitative determination of total 25(OH)D (including 25(OH)D₂ and 25(OH)D₃) in human serum or plasma (lithium heparin, K₂-EDTA). For this study, fasting venous blood samples will be collected, allowed to clot at room temperature, and centrifuged at 3,000 × g for 10 min to obtain serum, which will be stored at −80 °C until analysis.

From an analytical perspective, the assay demonstrates a functional sensitivity of 4.01 ng/mL (10.03 nmol/L), with a CV of 18.5%. The response is linear across the full measuring range; on the Cobas e 601 platform, analytically comparable to the Cobas Pure analyser, linearity has been verified between 1.67 ng/mL (4.18 nmol/L) and 133 ng/mL (332.5 nmol/L), with deviations from linearity consistently below ± 2 ng/mL (5.0 nmol/L) or 10% bias.

According to the manufacturer’s validation data, the Elecsys® Vitamin D total III assay, when performed on the Cobas Pure automated immunoassay analyser, has a Limit of Blank (LoB) of 2.0 ng/mL (5.0 nmol/L), corresponding to the highest measurement expected in the absence of analyte. The Limit of Detection (LoD) is 3.0 ng/mL (7.5 nmol/L), defined as the lowest concentration that can be distinguished from the LoB with 95% probability. The Limit of Quantitation (LoQ) is 6.0 ng/mL (15.0 nmol/L), representing the lowest concentration measurable with acceptable precision, defined as a coefficient of variation not exceeding 20%.

To evaluate the vitamin D status of each patient, we will adopt the 25-OH-D cut-off values proposed by SIOMMS (56). The severe deficiency will be reported for 25-OH-D < 10 ng/mL levels (<25 nmol/L); deficiency for 25-OH-D between 10 and 20 ng/mL (25–50 nmol/L) levels; insufficiency for 25-OH-D between 20 and 30 ng/mL (50–75 nmol/L) levels; adequacy for 25-OH-D between 30 and 100 ng/mL (75–125 nmol/L) levels and toxicity for 25-OH-D > 100 ng/mL (>125 nmol/L) levels (57).

### 2.4 Statistical analysis

#### 2.4.1 Sample size calculation

Regarding the background for the sample size calculation, to identify a small number of miRNAs to be validated as diagnostic MS-associated miRNA profiles in RT-qPCR on serum samples, a bioinformatic analysis was preliminarily conducted and described in section 2.2.3 Internal exposome variables.

As for the sample size calculation, to validate variation among the selected MS diagnostic miRNA, in response to urban air pollution, urbanisation, lifestyle and QoL components of the external exposome, the differential expression (ΔCT) for each miRNA will be considered as the endpoint measure. To compare the mean difference of ΔCT for each miRNA among exposure groups and to include the possible modulating effect in terms of accommodating covariates, negative binomial generalised linear models (GLM) will be used. Sample size has been calculated accordingly (58) through an estimating procedure based on a representative exemplary dataset created for a single miRNA comparison among two groups of pwMS defined by a specific external exposure variable and including a vector of covariate variables for the miRNA sample. The alternative hypothesis that the logarithm of miRNA fold change is different from zero against the null hypothesis of a log fold change equal to zero, by the likelihood ratio test statistic, will also be tested.

The minimum number of pwMS necessary to achieve 80% of power, considering a desired minimum miRNA fold change between 1.1 and 4.5, was 168. Therefore, 200 eligible pwMS who meet the inclusion criteria and sign the informed consent will be included in the study, considering a 15% dropout at the blood sampling stage.

#### 2.4.2 Feasibility of the study and structure of exposomic data

Due to the high number of variables selected for EXPOSITION, we tested the information content of the variables for each exposomic component and their integration with correlated variables. Factor analysis was performed using a hybrid dataset (real data already collected by the researchers and synthetic data modeled on the real dataset), to explore how different exposures may cluster into common latent factors, whether the data may show sufficient correlation/structure to be meaningfully summarized, and how dimensionality can be reduced while preserving relevant information. The analysis performed on synthetic data indicated that the selected variables of the external exposome can cluster into 5 latent factors, which appear biologically and clinically plausible in the context of Multiple Sclerosis. These findings support the creation of exposure indices for subsequent informative analyses in the real dataset. To illustrate the analytical framework, we provide additional details in the Supplementary materials.

#### 2.4.3 Data pre-processing, integration and missing imputation

Social, demographic, clinical, internal and external exposome individual data from the sample of pwMS relevant to research objectives, in line with local and European regulatory requirements, will be stored within a unified EXPOSITION database and linked using the individual pseudonymized code created for each pwMS. Air pollution and urbanisation data will also be linked. Specifically, pwMS will be assigned the ecological characteristics (e.g., pollution gridded emission) of the geographic unit (municipality) where they are residents. Candidate and validated biomarker’s distribution will be evaluated. Variables with skewed distributions will be transformed (e.g., log-transformation) as appropriate. Values below the limit of detection (LOD) will be handled using standard procedures (e.g., substitution with LOD/√2) or multiple imputation, depending on the proportion of censored values. Missing values will be addressed using multiple imputations under the missing-at-random assumption, generating several imputed datasets that will be analysed separately and combined using Rubin’s rules.

All the analyses will be performed with the R statistical software, using the most up-to-date packages and pipelines. We will use Bioconductor, R’s development project, for the analysis and interpretation of high-throughput genomic data, which currently comprises more than 900 interoperable packages.

#### 2.4.4 Data description

The first round of statistical analyses will be used to describe the pwMS sample and internal and external exposome. Descriptive statistics will be performed with mean, standard deviation, median and interquartile range, or frequencies depending on the nature of the variables. Graphical descriptions will be used by boxplots or violin plots, with the option of grouping with the categorical exposures.

#### 2.4.5 Exposome clustering, correlation and association analyses

a) *Clustering.* Clusters of individuals who share similar exposure patterns will be identified using hierarchical clustering.
b) *PCA.* Principal component analysis will be used to reduce the dimensionality of the exposome (e.g., PCA standard, robust or FAMD for both categorical and continuous exposures).
c) *Assess correlation structure.* Pearson, Spearman or Cramer coefficients, as appropriate, will be calculated to determine correlation structures between variables of the same exposome component. A correlation matrix and heatmap or Circos plot will be created.
d) *Validated biomarkers’ behaviour in the pwMS sample (Neurofilament proteins, levels of pro- and anti-inflammatory cytokines, vitamin D level).* They will be tested for correlation with the miRNA relative expression and with alpha, beta diversity and relative abundances; they will also be tested for association with each exposure variable of the external exposome.
e) *Single exposure association analysis with the primary endpoint.* To estimate the association between the external exposome and the expression of miRNAs (the primary endpoint), separate regression models (negative binomial, linear, or mixed-effects, depending on the outcome distribution) will be fitted, taking account of previous clustering, and adjusting for confounders and other modifiers of effect (including the validated biomarkers described above).
f) *Single exposure association analysis with the secondary endpoints.* Gut and nasal microbiota data, as the secondary endpoint, will be analysed employing non-parametric statistical tests (e.g., Mann–Whitney, Kruskal-Wallis tests) for comparing alpha-diversity and relative abundances, whereas a permutational multivariate analysis of variance with F-ratios (“adonis”) will be used for beta-diversity comparison. Linear regression models will be employed to perform multivariate statistics on biodiversity and relative abundances, correcting estimates for confounders and modifiers of effect (including DMT type and dietary habits as MEDI-LITE and DII).
g) *Apply multiple testing correction.* For each association tested, both raw *p*-values and FDR (Benjamini–Hochberg) q-values will be reported; if exposures are strongly correlated, the ENT-Bonferroni p-value will also be reported (59–61)
h) *Variable selection (e.g., exposures).* The modelling procedure will identify a final set of predictors of miRNAs or microbiome variation, allowing for the simultaneous inclusion of multiple exposures within the same model. Variable selection will be performed using generalised multivariable linear models estimated via penalised maximum likelihood, applying either a lasso or elastic net penalty to determine the most informative exposures associated with the outcomes.

## 3 Discussion

The EXPOSITION study protocol represents a pioneering effort to elucidate the intricate interplay between the external exposome and its effects on OS, inflammation, and overall health, particularly in individuals with MS.

Our sample, drawn from Pavia and Milan, is intentionally aligned with the NBFC’s multi-city design (62): these sites serve as Northern Italy anchors to characterise urban exposome–health relationships within the Po Valley.

By adopting the exposome framework, this research aims to broaden the scope of understanding and prevention of various diseases, moving beyond the traditional approach of environmental epidemiology.

Conventional environmental epidemiology has primarily focused on external environmental exposures, such as pollutants and physical agents; in contrast, the exposome approach captures the totality of exposures, encompassing both external factors and internal components such as specific biomarkers, genetic predispositions, epigenetic modifications, and metabolic profiles (16, 60, 61). This holistic perspective enables the identification of previously unrecognised associations, deepens understanding of disease aetiology, and facilitates the discovery of novel biomarkers of exposure (60, 61). Unlike the hypothesis-driven investigations that dominate environmental epidemiology—often focusing on a single environmental factor and its direct relationship to a health outcome—the exposome approach addresses the multifactorial nature of most diseases (11, 63). Indeed, by integrating distal risk factors from the external environment with proximal influences from the biological internal environment, this methodology seeks to unravel potential interactions and causal mechanisms underlying diverse health outcomes (25, 60, 61).

The EXPOSITION study employs innovative methodologies to evaluate a wide range of environmental exposures, alongside state-of-the-art biomarker analyses, to explore the environmental contributions to MS pathophysiology.

Understanding how these exposome factors influence MS is vital for uncovering mechanisms that drive the disease and for advancing therapeutic and preventive strategies. Exposome factors, spanning environmental, occupational, and lifestyle influences, have significant implications for immune function, neuroinflammation, and CNS integrity.

By examining these interactions, the study aims to identify modifiable risk factors that contribute to the onset and progression of MS.

For instance, the role of the Epstein–Barr virus in eliciting aberrant immune responses, the impact of vitamin D deficiency on immune modulation, and the exacerbation of OS and inflammation by air pollutants such as PM2.5 all highlight specific pathways for therapeutic intervention (11, 14, 64). These insights could lead to targeted strategies, such as antioxidant therapies, pollution-control policies, and personalised lifestyle interventions. Additionally, understanding the mechanisms by which exposome factors compromise the blood–brain barrier or promote neurodegeneration could pave the way for therapies aimed at preserving CNS integrity and halting disease progression. This approach also facilitates patient stratification based on exposure profiles, enabling the development of more personalised prevention and treatment plans.

The anticipated endpoints of the EXPOSITION study are expected to significantly expand our understanding of the environmental triggers of MS and provide a foundation for developing targeted interventions and precision medicine strategies. By focusing on modifiable environmental factors, these approaches have the potential to curb disease progression, ultimately improving the QoL for individuals with MS. The study’s findings could also inform broader public health policies, emphasising the importance of addressing environmental exposures to reduce the overall burden of MS. Furthermore, the methodologies employed in this study could be adapted for investigating other NCDs, thereby extending their impact beyond MS.

The results are also anticipated to empower patients by elucidating the extent to which external exposome factors influence MS, fostering better disease comprehension.

Additionally, the findings will provide actionable evidence to policymakers, supporting the development of interventions targeting external exposome through healthcare systems and related organisations; this could include strategies such as public health initiatives, urban planning adjustments, and institutional measures designed to mitigate harmful environmental exposures.

Despite its groundbreaking approach, the EXPOSITION study has certain limitations.

First, it will be explicitly acknowledged that the current results will not establish national representativeness and will be interpreted as northern benchmarks pending aggregate NBFC analyses (62) in other Italian cities. Indeed, in the EXPOSITION project, (i) the geographical scope will be limited to two cities in Northern Italy, which could limit demographic and environmental representativeness, and (ii) the specific meteorology of the Po-Valley (e.g., atmospheric stagnation, thermal inversions) could amplify exposure contrasts compared to other regions.

Second, a key limitation is the absence of a comparator (non-pwMS) group; by design, the study explores exposome-biomarker heterogeneity within pwMS rather than differences between pwMS and the broader population. Consequently, we cannot determine whether the observed biomarker-exposome relationships are MS-specific or reflect general patterns present in non-MS individuals. This constrains external validity and the specificity of inference. Future work will include non-MS comparators from the general population to directly test disease specificity and enhance the generalizability of the findings.

Last, the scope of variables analysed is constrained, and from a molecular perspective, transcriptomic analysis is restricted to circulating RNA from collected biological samples. Due to the invasive nature of tissue collection, the regulatory role of miRNAs on specific target mRNAs remains speculative, inferred through prior bioinformatic analyses and literature review rather than direct tissue validation. These constraints highlight areas for future research to build upon the study’s findings.

In summary, the EXPOSITION study holds the potential to make a substantial contribution to MS research by elucidating the disease’s environmental determinants and opening avenues for innovative interventions. To strengthen this line of inquiry, future work should directly validate tissue samples to further explore the regulatory role of miRNAs and incorporate a broader set of exposure variables and biomarkers. This comprehensive approach may not only enhance precision medicine for MS but also serve as a model for studying other complex diseases, ultimately advancing both science and public health.
