# Supplementary material for: Exposome, oxidative stress and inflammation in persons with multiple sclerosis: the EXPOSITION study protocol
Source: Front Public Health. 2025 Oct 22;13:1688158. doi: 10.3389/fpubh.2025.1688158 (PMC12586111; doi:10.3389/fpubh.2025.1688158)
Supplement: Supplementary file 1 [file Supplementary_file_1.docx]

**Supplementary Material**

**Identifying common latent structures among External Exposome variables using Explorative Factor Analysis.**

**Methods**

1. **Building the hybrid dataset**

150 subjects from the EXPOSITION real dataset were included and selected 59 variables (including external exposome factors and demographic and clinical data). We generated a synthetic sample of n=100 observations including the same variables using the CART (Classification and Regression Trees) method, implemented in the R synthpop library [1], which is particularly suitable for our dataset with both categorical and continuous variables. Real and synthetic data were combined in a hybrid dataset with 250 statistical units.

1. **Data preprocessing and Factor Analysis (FA)**

The hybrid dataset underwent a pre-processing phase (assessment of normality, replacement of undetectable values with LOD, graphical explorations) and for missing values, we applied multiple imputation using the R mice library with parameters m=10 and maxit=50 [2].

Following confirmation of factorability (Kaiser–Meyer–Olkin (KMO) measure of sampling adequacy and Bartlett’s test of sphericity), the number of factors (F) suggested was determined through Parallel Analysis. Factor extraction was subsequently performed using the ‘psych’ package in R [3] and orthogonal rotation executed using the varimax procedure, as implemented in the ‘GPArotation’ package.

1. **Results**

We performed an exploratory FA on the exposure variables defining the general and specific external exposome of a hybrid dataset.

The KMO measure confirmed sampling adequacy, and Bartlett’s test of sphericity was significant, supporting the suitability of the data for FA. Parallel analysis yielded a five-factor solution: after Maximum Likelihood extraction and Varimax rotation, a simple and interpretable factor structure emerged (Figure S1).

Factor 1 (LM1) was defined by high loadings on NO2 short-term mean, NO2 long-term mean, PM2.5 short-term mean, PM2.5 long-term mean, Vitamin D levels in plasma representing, mainly, the Air Quality dimension. Factor 2 loaded strongly on Weight, BMI, Waist circumference, Waist-to-Height Ratio, corresponding to Body Composition dimension; Factors 3–5 reflected clustering in dimensions we classified, respectively as Physical Functional Status, Diet and Inflammation, Aging and Lifestyle. The results suggest an underlying structure that captures the main dimensions of external exposures. The five-factor solution explained 41% of the cumulative variance; however, since the purpose of this analysis was to assess methodological feasibility rather than to establish a definitive factor structure, the variance explained was considered acceptable for illustrative purposes.

**References**

[1] Nowok, B., Raab, G. M., & Dibben, C. (2016). Synthpop: Bespoke Creation of Synthetic Data in R. Journal of Statistical Software, 74(11), 1–26. <https://doi.org/10.18637/jss.v074.i11>

[2] van Buuren, S. (2018). Flexible Imputation of Missing Data, Second Edition (2nd ed.). Chapman and Hall/CRC. <https://doi.org/10.1201/9780429492259>

**Figure S1.** Identifying common latent structures among External Exposome variables using Explorative Factor Analysis.


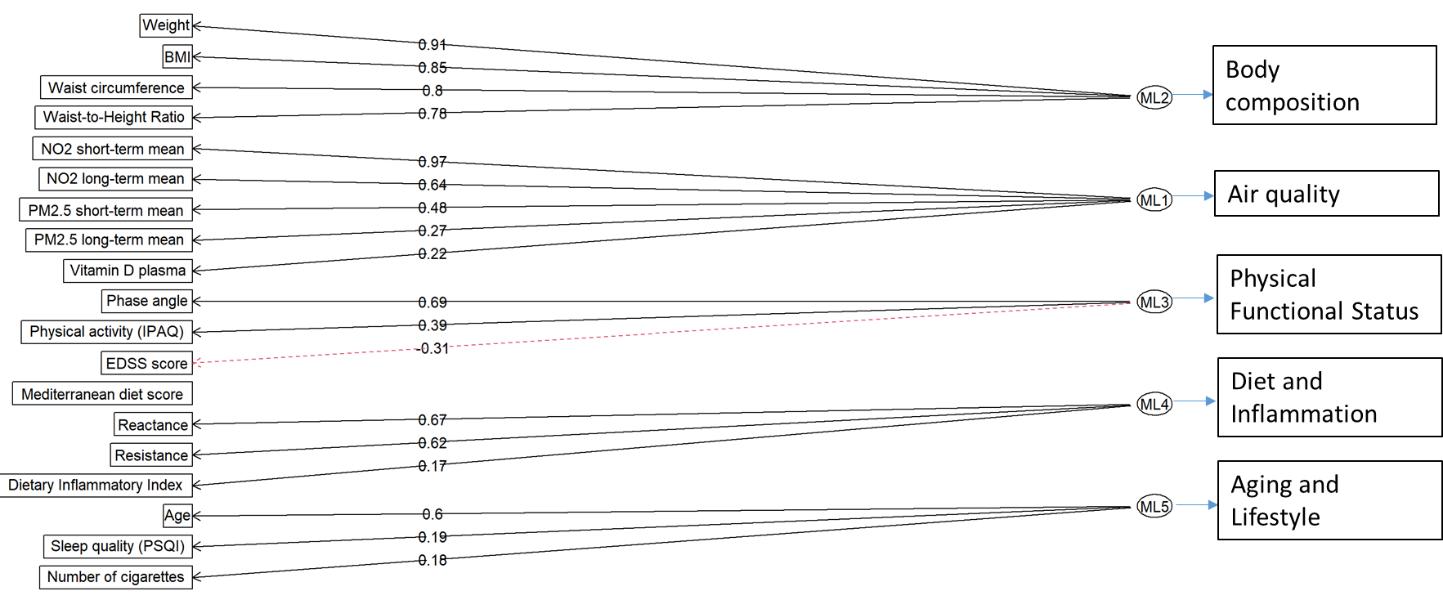


**Legend.** ML: Maximum Likelihood Factor; BIA: Bioelectrical impedance analysis.

Variance explained by FA: 41%
